# Influence of socio-demographic factors on awareness of HIV/AIDS among Bangladeshi garment workers

**DOI:** 10.1186/2193-1801-2-174

**Published:** 2013-04-20

**Authors:** ATM Hasibul Hasan, Rashedul Hassan, Zillur Rahman Khan, Elham Nuzhat, Uditi Arefin

**Affiliations:** Dhaka Medical College Hospital (Medicine OPD), Dhaka, Bangladesh; Shaheed Suhrawardi Medical College Hospital (Medicine), Dhaka, Bangladesh; Child and Adolescent Psychiatry, National Institute of Mental Health, Dhaka, Bangladesh; Department of Paediatrics, Mymensingh Medical College Hospital, Mymensingh, Bangladesh; Department of Endocrinology, Holy Family Red Crescent Medical College Hospital, Dhaka, Bangladesh

**Keywords:** HIV/AIDS, Garment workers

## Abstract

The study was conducted to assess the level of awareness on HIV/AIDS (Acquired Immune Deficiency Syndrome) and the Influence of different socio-demographic factors among the garment workers in Bangladesh. This cross sectional study was carried out among 303 workers in three selected garment factories in Dhaka city from July 2010 to June 1011. Data were collected by face to face interview through a predesigned questionnaire containing desired information. The majority of workers (76.6%) were within the 17–19 years age group. The female respondents predominated (55.1%). A considerable number of the sample population (39.3%) completed the primary education. But majority belonged to low income group (68.4%), followed by the very low income group (22.4%). Almost everyone (98.3%) except five of the respondents heard the word HIV/AIDS and most of them (90.6%) knew that the disease is transmissible from person to person and mainly by sexual intercourse (78.9%). Only 28.2% had some idea about the sign symptoms of HIV. About (64.4%) thought that persons having heterosexual partners (including prostitutes) are high-risk groups. Though many (74.2%) of the respondents thought that HIV/AIDS is preventable, only 45% said HIV/AIDS is not curable. But 70.5% answered that death is the ultimate fate. The main source of information was radio/TV, newspaper. Unfortunately, 76.9% of the respondents had poor awareness while only 10.6% had good awareness. The level of awareness increased with age (*p* = <0.05). Though the male were slightly more aware than the female, the relationship is not statistically significant (p= > 0.05). Awareness among S.S.C. passed and above is quite more than the awareness of illiterate (p = <0.01). But there was no relation (p= > 0.05) of level of family income and living pattern with level of awareness. Even being a risk group the garment workers not much aware of HIV/AIDS. The level of awareness increased with age and literacy, which shows the window of opportunity for the policymakers that educational intervention program, may be effective for them.

## Introduction

Since discovery of AIDS in 1981 and its causative agent in 1983–1984, there have been significant advances in our understanding of this disorder (Salam and Yousuf 1995). The etiologic agent, human immunodeficiency virus (HIV) which cause destruction of helper T-lymphocytes leading to the development of a cellular immunodeficiency disease rendering the patient susceptible to a wide variety of opportunistic infections (Salam and Yousuf 1995). Once AIDS is diagnosed, a person in a developing country may not survive for more than 2 years (SEARO 2000). HIV is transmitted principally through unprotected sexual intercourse, heterosexual or homosexual and also through blood or blood products, donated semen or organs, or from an infected mother to her child. More than 80% of infections in the South-East Asia Region are a result of heterosexual transmission (SEARO 2000). As there is no vaccine, primary prevention is the only tool to control HIV infection and AIDS (Salam and Yousuf 1995). AIDS is increasingly becoming a major public health concern in many developing countries (Khan 2002). Bangladesh is fortunate to have a low HIV/AIDS prevalence of less than 0.1% (UNICEF 2009). However, the threat is significant for Bangladesh, considering the factors like geographic location, large number of population with STD (Sexually Transmitted Disease) cases and above all, lack of proper knowledge about safe sex among the people. Female adolescents are more vulnerable than any other and are biologically more susceptible to HIV infection (Khan 2002).

Bangladesh being a developing country, in the economic sector, the newly emerged garments industries have been creating job opportunities to a large number of people in the cities. It now ranks among the largest garments exporters of the world which accounts for 75% of the foreign currency and 25% of GDP of Bangladesh (Mottaleb 2011). Most of the garments workers are female and majority of them are adolescent and unmarried (Jahan 2012). The literacy rate being low, these workers are not informed about menstruation, safe sex, contraceptive methods, STDs, and HIV infection. As there is large scale of social insecurity, adolescent garments workers are often victims of terrible sexual abuse. So, the adolescent garments workers are a vulnerable group for HIV infection (Jahan 2000). Awareness of AIDS among adolescents is yet to be explored. It is imperative to understand how much of female adolescents know about AIDS and fatality and how to prevent the disease (Khan 2002). So we conducted this study to ascertain the existing level of awareness about HIV/AIDS including its mode of transmission and preventive measures among these garments workers from some selected factories in Dhaka City. We also tried to evaluate the relationship between the level of awareness and different socio demographic factors.

## Material and methods

It is a cross sectional type of descriptive study conducted among the workers from three selected garments factories in Dhaka city from July 2010 to June 2011. The garments were selected by purposive sampling technique which was governed by easy communication, assurance of cooperation and above all the consent from the workers. The respondents were randomly selected from their working place. The study was conducted up on 303 garment workers between the age of 10–19 years in 3 different selected garments factories situated in Dhaka city. The data collected by a semi-structured questionnaire containing socio-demographic information (age, sex, education, income) and 14 questions related to knowledge of HIV/AIDS with 42 probable answers (see information check list section). Each correct answer carried 1 mark and wrong or unknown answers carried 0 mark. Thus the respondents were given score between 0–42. The level of awareness was measured by arbitrarily ranking the score. A score above 21 was considered good awareness (i.e. a respondent giving at least 50% correct answers), from 17–21 (40–49% correct answer) was labeled as average awareness and those below 17 (<40% correct answer) as poor awareness. The questionnaire was pre-tested among garments workers of a selected garments factory and necessary modification and correction had been done prior to final stage of data collection. The study protocol was approved by the ethical review committee of Dhaka Medical College. Informed written consent was taken from each of the respondents. Information was collected from the respondents through face to face interview of 15 to 20 minutes.

Statistical analyses of the results were obtained by using window based computer software devised with Statistical Packages for Social Sciences (SPSS-16) (SPSS Inc, Chicago, IL, USA). The results were presented in tables, figures and diagrams. During analysis frequency distribution for all the variables were worked out and produced in tabular form. The χ^2^ tests were used to compare proportions. A two-tailed *p* value of 0.05 was considered significant at 95% CI (Confidence Interval) level.

### Information check list

I. **Socio-demographic variables:**
  1. Age
  2. Sex
  3. Marital status
  4. Education
  5. Monthly income:
    - Personal income
    - Family income
  6. Housing condition
  7. Family size
  8. Living with family or not
II. **Variables related to AIDS knowledge:**
  1. Causative agent
  2. Route of transmission
  3. Sign & symptom
  4. Methods of prevention
  5. Place to go for treatment
  6. Ill effects of AIDS
  7. Risk group

### Operational definitions

1. **Educational status:** It is classified into following groups:
  A. **Illiterate:** The respondents who can neither read nor write.
  B. **Non-formal education:** The respondents who can read or write but have not attended schools or colleges.
  C. **Primary:** The respondents who have attended the school upto class V.
  D. **Lower secondary:** The respondents attending the school between class V to class VIII.
  E. **Secondary:** The respondents attending the school above class VIII to S.S.C. level.
  F. **Higher secondary:** The respondents above S.S.C. level upto H.S.C. level.
2. **Family income:** It is the total amount of money earned by the respondent and by his/her family members in each month. It is classified as follows:
  A. **Very low income:** Family income upto 4000 Tk per month.
  B. **Low income:** Monthly family income is in between 4001–10000 Tk.
  C. **Moderate income:** Monthly family income is in between 10001–20000 Tk.
  D. **Good income:** Monthly family income is more than 20000 Tk.
3. **Mess:** Group of people living in the same residence and regularly taking meals together.
4. **Boarding house:** It is a house where a paying guest is provided with meals and lodging.
5. **Awareness scoring:** Total 42 answers, each correct answer carries 1 and wrong answer, do not know or others carry 0.
6. **Awareness grading:**
  A. **Good awareness:** Correct answer ≥ 50% i.e. awareness score is 21 or above.
  B. **Average awareness:** Correct answer 40 to 49% i.e. awareness score is 17 to 20.
  C. **Poor awareness:** Correct answer < 40% i.e. awareness score is below 17.

## Result

This cross-sectional study involved 303 respondents. The majority of them, (76.6%) were within the 17–19 years age group. A minor proportion, (2%) were under 14 years. The rest of the respondents, 65 (21.5%) were in 14–16 years group. The female respondents predominated (55.1%) in this study. A considerable number of the sample population (39.3%) completed the primary education, many went to high school (30.7%), but only 9.9% passed secondary certificate examination and 1.6% passed higher secondary exam. About 16.8% were illiterate. On the basis of monthly family income majority belonged to low income group (62.2%), followed by the very low income group (22.4%). Many of the workers lived with their families (58.7%). Among the rest (41.3%), a good number lived in mess (22.8%), some, with relatives (15.2%) and the others (3.3%) in hostels, boarding houses (Table 1). Almost everyone (98.3%) except five of the respondents heard the word HIV/AIDS. Among them 90.6% knew that the disease is transmissible from person to person and mainly by sexual intercourse (78.9%). More than one third of them also thought that multiple sex partner is a mode of transmission. Only 28.2% had some idea about the sign symptoms of HIV. Majority of them thought of fever (64.3%), some knew about fatigue (45.2%), weight loss (29.8%), and malaise (22.6%) as the feature of HIV. About (64.4%) thought that persons having heterosexual partners (including prostitutes) are high-risk groups. The others pointed out about multiple sex partners (39.9%), blood transfusion (21.8%), drug abusers (18.5%), bus or truck drivers or rickshaw pullers (11.4%) are the high risk groups. But 19.5% had no idea about the fact. Majority, 221 (74.2%) of the respondents thought that HIV/AIDS is preventable and mostly avoiding heterosexuality (including prostitutes) (71.9%), avoiding multiple sex (47.1%), using condom(43.4%), avoiding sharing of needles and syringes (38.0%), screening of donated blood (20.4%), preventing vertical transmission (6.8%). Interestingly 45% of the respondents said HIV/AIDS is not curable. But 70.5% answered that death is the ultimate fate. Majority, 246 (82.6%) heard about HIV/AIDS from radio/TV, 24 (8.1%) from newspaper, 35 (11.7%) from friends, 14 (4.7%) from books, 19 (6.4%) from health workers and 11 (3.7%) from organized AIDS education program. But 15 (5.0%) respondents could not remember the source. The study reveals that the awareness of majority of the respondents, 233 (76.9%) was poor while 38 (12.5%) had average awareness. Only 32 (10.6%) had good awareness (Table 2).

**Table 1:** **Socio demographic profile of the respondents (N = 303)**

Parameter	n	%
**Age**		
<14 yr	6	2
14–16 yrs	65	21.5
17–19 yrs	232	76.6
**Sex**		
Male	136	44.9
Female	167	55.1
**Level of Education**		
Illiterate	51	16.8
Primary	119	39.3
Lower secondary	93	30.7
Secondary	30	9.9
Higher secondary	5	1.6
Non formal	5	1.6
**Family Income**		
Very Low income	68	22.4
Low income	189	62.2
Moderate income	0	0
Good income	46	15.2
**Residence pattern**		
Living with Family	178	58.7
Living with others		
Mess	69	22.8
Relative	46	15.2
Boarding	04	1.03
Others	06	2

**Table 2:** **Awareness of the respondents regarding HIV/AIDS (N = 303)**

Parameter	n	%
**The Term HIV**		
Heard about it	298	98
Didn’t hear about it	5	2
**Disease Transmission**	**(n = 298)**	
**Transmissible**	270	90.6
Route *-		
Through sexual intercourse	213	78.9
Through blood transfusion	104	38.5
In intravenous drug abuser	35	13
Through equipment & needles	108	40
Mother to child transmission	54	20
Homosexuality	13	4.8
Multiple sex partners	101	37.4
Don’t know	12	4.4
Others	10	3.7
**Not Transmissible**	12	4
**Don’t know about it**	16	5.4
**Knowledge about symptoms**		
**Have some idea**	84	28
Symptoms *-		
Chronic diarrhea	9	10.7
Malaise	19	22.6
Fever	54	64.3
Rash	16	19
Sore throat	10	11.9
Fatigue	38	45.2
Severe weight loss	25	29.8
Night sweating	7	8.3
Others	19	22.6
**Don’t know about it**	214	72
**Knowledge about risk group**		
Persons having multiple sex partners	119	39.9
Homosexual & bisexual person	21	7
Heterosexual partners (including prostitutes)	192	64.4
Intravenous drug abusers	55	18.5
Transfusion recipients of blood & blood products	65	21.8
Long route bus & truck drivers and rickshaw pullers	34	11.4
Don’t know	63	19.5
Others	12	4
**Knowledge on protection**		
Know about protection*	221	74.2
Using condom	96	43.4
Avoiding multiple sex	104	47.1
Avoiding homosexuality and bisexuality	13	5.9
Avoiding heterosexuality	159	71.9
Avoiding sharing of needle and syringe	84	38
Screening of donated blood	45	20.4
By preventing transmission (mother to child)	15	6.8
Discouraging sharing of tooth brushes and razors	8	3.6
Others	18	8.1
**Not preventable**	27	9.1
**Don’t know about it**	50	16.1
Fate of HIV/AIDS infection*		
Curable	134	45
Death	210	70.5
Don’t know	53	17.8
Others	35	11.7
Source of Information*		
Radio/TV	246	82.6
Newspaper	24	8.1
Books	14	4.7
Friends	35	11.7
Health worker	19	6.4
Organized AIDS education program	11	3.7
Can’t remember	15	5
Others	13	4.4
**Level of awareness**		
Poor	233	76.9
Average	32	12.5
Good	38	10.6

*Multiple responses.

There was an association between the age of the respondents and their level of awareness. The number of respondents in 17–19 years age group had better awareness compared to those less than 14 years of age. This was also statistically significant (*p* = <0.05). The study showed that, out of 136 male respondents, 18 had good awareness while among 167 female, 14 had good awareness. That is, male were slightly more aware than the female, though their relationship is not statistically significant (*p*= > 0.05). Awareness among S.S.C. passed and above is quite more than the awareness of illiterate (*p* = <0.01). But there was no relation (*p*= > 0.05) of level of family income and living pattern with level of awareness (Table 3).

**Table 3:** **Relation of level of awareness with socio-demographic variables**

Variables	Poor	Average	Good	***p*** value
**Age**				
<14 yr	5	1	0	
14–16 yrs	60	2	3	<0.05
17–19 yrs	168	35	29	
**Sex**				
Male	98	20	18	>0.05
Female	135	18	14	
**Level of Education**				
Illiterate & Non formal	49	5	2	
Primary & High school	167	22	23	<0.01
SSC and above	17	11	7	
**Family Income**				
Poor	49	5	4	
Lower Middle Class	144	25	20	>0.05
Upper Middle Class	24	7	8	
Higher	6	1	0	
**Residence pattern**				
Living with Family	132	25	21	>0.05
Living with others	101	13	11	

## Discussion

This study had been designed to inquire the awareness about HIV/AIDS among the young vulnerable garments workers on the basis of the global threat that the human era facing these days. To date little is known about awareness of HIV/AIDS among adolescent garments workers in Bangladesh. Our aim was to explore the awareness of HIV/AIDS among adolescent garments workers, who are quite neglected section of the society with regards to access to information and services.

Among the respondents of this study majority were in 17–19 years of age group, which is in agreement with (Khan 2002), (Jahan 2000), (Perez and Dabis 2003), (Goel and Pandey 1997). With increasing age, adolescents are exposed to new experiences relating to sexuality and reproduction (Khan 2002). Interestingly, they had relatively good awareness on HIV/AIDS than in <14 years age group and the relation was also statistically significant (*p* = <0.05). Such degree of poor awareness in the later group (<14 year) may be due to the fact that traditional social system and health care service often ignore this group as a separate entity who are treated neither as child nor as adult. Their age remains the main barrier of communication with adults regarding information on sex and sexually transmitted diseases like HIV/AIDS. This is also in close agreement with Bangladesh Demographic and Health Survey (BDHS) (Khan 2002), which reported that only 7.8% in 10–14 year age group are aware about AIDS. But in more matured group (15–19 years), 18.3% are aware of the same fact. In this study the ratio of male and female respondents were almost equal. This is inconsistent with (Jahan 2000), (Paul-Majumder 2008) and reports from electronic and print media. The later reported that 90% of workers are female (Nafiz 2009), which is due to appropriateness of the job and sincerity of women. But in this study male and female were equally chosen to make a comparison of awareness with sex distribution. Although the relationship of awareness with distribution of sex is not statistically significant, the male were found to have slightly better aware than the female which is not in agreement with study of research and evaluation division of BRAC (Ara and Karim 1999). The level of awareness was good only in a few of them (10.6%) whereas the majority was poor regarding the awareness on HIV/AIDS, which is not quite unusual in the perspective of Bangladesh. Education positively contributed to the awareness on HIV/AIDS which was also statistically significant in this study (*p* = <0.01). It is in agreement with (Goel and Pandey 1997). They showed that AIDS awareness was much greater in St. Mary’s college students than other students in Meghalaya, where previous lectures on this topic were delivered. It reflects the positive influence of literacy for improvement of knowledge (Jahan 2000). (Khan 2002), (Jahan 2000) and (Mondal et al. 2008) also reported the similar associations. This can be explained by the fact that educated people can acquire more knowledge when they are exposed to source of available information like electronic media (Computer, internet), printed papers (Books, newspapers, posters, booklets). A large portion of the respondents had low socioeconomic background, which had forced them to work at this age. But the family status revealed no significant relationship with their level of awareness. On socioeconomic and cultural background of our country, people learn their norms and values of life while growing up in family environment at home which is also evident from the finding of this study that those living with the family had slightly greater knowledge than the rest. Among the rest, those living in mess have the opportunity to share their views and ideas with their mates and so they are more aware of the fact.

In this study almost all the respondents said that they have heard about HIV/AIDS. This is very much similar with (Goel and Pandey 1997), (Klepp and Lugoe 1999) in Tanzania. While comparing the knowledge on HIV/AIDS (Khan 2002) showed that when 17% of adolescent of Bangladesh had ever heard of AIDS; Indians and Nepalese reported 37% and 25% respectively. His study was reflected on the general adolescent population of these countries, which is not similar with our study as because we have concentrated on a group of population in Dhaka city who are provided with more facilities to get information from mass media. Mass media exposure such as Radio and TV was positively associated with having knowledge on HIV/AIDS among the adolescent. The exposure to such media can communicate knowledge on AIDS in music, news reports, songs, dramas, documentaries and advertising and can profoundly influence attitudes and behavior of people (Khan 2002). The splendid contribution of mass media is also shown in a mass media project using a TV program to teach adolescent in Zaire about HIV/AIDS awareness (Convisser 1992). Regarding the source of information, Radio and TV were reported as a major source of information on HIV/AIDS by our respondents. This is quite similar with (Goel and Pandey 1997) and (Khan 2002). More over 21% of the respondents in our study, cited that they first came to know about HIV/AIDS from the health personnel. This shows the effectiveness of various programs implemented by the Govt. of Bangladesh in association with international and national NGOs in health sector.

Many had good idea about route of transmission. This is a good sign that only few have misconception about the transmission who thought that it might be transmitted by casual contact, shaking hands, eating together which shows the similarity with Goel N.K et al., conducting a study among the nursing professionals (Goel and Pandey 1997). This inspiring result is due to high coverage of mass media specially Radio and TV among the respondents. Many of the respondents were very much aware that this disease is a preventable one. But UNICEF reported that only 18% of male and 8% of female have comprehensive knowledge about prevention of HIV/AIDS among people aged 15–24 years (UNICEF 2009). (Singh and Joshi 2012) had reported high proportion of misconception (63%) among truck-drivers in India which is also due to same fact found by Klepp in Tanzania that the lack of exposure and poor coverage of AIDS related issue in mass media being the leading cause of poor awareness among Tanzanians previously (Klepp and Lugoe 1999). (Pandey et al. 2011) showed that intensive intervention program taken for high risk truck divers motivated them for safer sexual behavior. Although the respondents had good knowledge about prevention and transmission of HIV/AIDS, very few could cite about sign/symptoms. The cause behind it is that less emphasis has been given on sign/symptoms in mass media, which should be considered by the policy makers. We found that only less than half of the respondents could give the correct answer that the disease is not a curable one which is not in agreement with the study by N.P Goel and Pandey, among the college students of Meghalaya (Goel and Pandey 1997). Probably it may be due to the impact of level of education. But this is different with finding of T.K. Hartung in South Africa (Hartung et al. 2002) in which the respondents had same educational status. This difference is due to awareness developing program in South Africa where there is high prevalence of HIV/AIDS. It is very promising to find that 71% of the respondents were aware that “death” is the ultimate destiny of patients with HIV/AIDS. This is quite similar with the finding of (Khan 2002) and (Hartung et al. 2002). On this point of view, majority considered HIV/AIDS as a serious problem for Bangladesh.

We had some limitations in the study. Firstly, we selected the study places semi purposely. Secondly, the study result only from Dhaka city may not reflect completely the whole scenario in Bangladesh. Finally the sample size was also small in relation to the total number of garments worker in the country.

## Conclusion

The study reveals that most of the respondents think HIV/AIDS as a transmittable disease. But they had no or very little idea about the sign symptoms of HIV/AIDS. Two very important observations have explored out from the study that the awareness is directly proportional to the age and level of literacy. So, it could be proposed, to raise awareness against HIV/AIDS emphasis should be given on increasing the literacy rate and arranging and strengthening the campaign program against HIV/AIDS among the garments workers.

Considering the epidemiological characteristics of AIDS pandemic in Asia, it is essential that the youth be given appropriate information and education on reproductive health matters including HIV/AIDS. There is a need for developing and implementing working group based program on HIV/AIDS prevention to reduce HIV related risk behaviors, particularly the unprotected sex. Mass education by a series of interventions and events at industry level, backed by effective interpersonal communication as peer education, factory based teaching and community action should be more extensively considered in this context.
